# Foam sclerotherapy versus ambulatory phlebectomy for the treatment of varicose vein tributaries: study protocol for a randomised controlled trial

**DOI:** 10.1186/s13063-019-3398-0

**Published:** 2019-07-03

**Authors:** Amjad Belramman, Roshan Bootun, Tristan R. A. Lane, Alun H. Davies

**Affiliations:** 1 Section of Vascular Surgery, Department of Surgery and Cancer, Charing Cross Hospital, Imperial College London, London, UK; 2 Specialty Training Registrar in Vascular Surgery, East of England Deanery, UK; 3 Specialty Training Registrar in Vascular Surgery, London Deanery, UK; 4 Imperial College Healthcare NHS Trust, London, UK

**Keywords:** Phlebectomy, foam sclerotherapy, superficial venous disease, varicose veins, varicosity treatment

## Abstract

**Background:**

Ambulatory phlebectomies and foam sclerotherapy are two of the most common treatments for varicose vein tributaries. Many studies have been published on these treatments, but few comparative studies have attempted to determine their relative effectiveness.

**Methods/design:**

This is a prospective single-centre randomised clinical trial. Patients with primary truncal vein incompetence and varicose vein tributaries requiring treatment will be assigned randomly to either ambulatory phlebectomies or foam sclerotherapy. The primary outcome measure is the re-intervention rate for the varicose vein tributaries during the study period. The secondary outcomes include the degree of pain during the first two post-operative weeks and the time to return to usual activities or work. Improvements in clinical scores, quality of life scores, occlusion rates and cost-effectiveness for each intervention are other secondary outcomes. The re-intervention rate will be considered from the third month.

**Discussion:**

This study compares ambulatory phlebectomies and foam sclerotherapy in the treatment of varicose vein tributaries. The re-intervention rates, safety, patient experience and the cost-effectiveness of each intervention will be assessed. This study aims to recruit 160 patients and is expected to be completed by the end of 2019.

**Trial registration:**

ClinicalTrials.gov, NCT03416413. Registered on 31 January 2018.

**Electronic supplementary material:**

The online version of this article (10.1186/s13063-019-3398-0) contains supplementary material, which is available to authorized users.

## Background

Approximately one-third of the adult population have varicose veins [1]. Chronic venous disease causes a significant negative effect on the quality of life (QoL) of patients; however, there is a significant improvement in the QoL following treatment for varicose veins [2–4].

Over the last 15 years, minimally invasive endovenous techniques for treating varicose veins have been introduced. These techniques have been proved to be cost-effective and safe, particularly when performed under a local anaesthetic in an outpatient setting [5]. The American Venous Forum and the National Institute for Health and Care Excellence have recommended endovenous thermal ablation techniques, namely radiofrequency ablation or endovenous laser ablation, as the first-line treatments for truncal reflux since 2011 and 2013, respectively [6, 7].

However, the treatment of prominent varicosities can be undertaken concurrently or at a later date using either ambulatory phlebectomy (AP) or foam sclerotherapy (FS) [6, 7]. Carradice and colleagues compared the concomitant treatment of varicosities to delayed treatment using AP following endovenous laser ablation of truncal veins [8]. Their data demonstrated that one patient (4%) in the concomitant group needed to have a re-intervention at 6 weeks compared to 16 out of 24 in the delayed treatment group [8].

Moreover, Lane et al. conducted a prospective comparative randomised trial comparing delayed AP against simultaneous AP after radiofrequency ablation. Their results showed that 2% of patients in the simultaneous group needed another intervention compared to 36% in the delayed group (*P* < 0.001) [9]. The relative risk that patients would require further varicosity treatment was 18.36, while the odds ratio was 27.78. In recent work by Gibson et al. with 50 patients, 65% of the patients required adjunctive treatment after 3 months of treating truncal veins with cyanoacrylate [10].

De Roos et al. compared sclerotherapy and AP [11]. They found that the recurrence rate at 1 year following sclerotherapy was 25% compared to 2.1% in the phlebectomy group. The relative risk for recurrence at 1 year was 12 and the odds ratio was 15.67. After 2 years, the recurrence rate was 37.5% in the sclerotherapy group with a relative risk of 18 and odds ratio of 28.20 [10]. The study, however, considered only the lateral anterior vein and used a liquid sclerosant rather a foam sclerosant, so that these results cannot be generalised to tributaries treated after truncal vein ablation using FS.

We, therefore, propose to undertake a randomised study comparing FS with AP to treat varicose vein tributaries concurrently with endovenous truncal ablation. The study is designed as a parallel group superiority study.

### Objectives

The primary objective is to ascertain the re-intervention rate following treatment with each modality. Pain score over the first 2 weeks will be assessed as a secondary objective using a 100-mm visual analogue scale and a numerical scale from 0 to 10. As additional secondary objectives, we will assess QoL, Venous Clinical Severity Score (VCSS), degree of bruising and phlebitis, patient satisfaction, time to return to normal activities and cost-effectiveness.

## Methods/design

### Study setting

Eligible patients will be recruited from Charing Cross Hospital (Imperial College London, UK).

### Eligibility criteria

Inclusion criteria are primary truncal vein incompetence and varicose vein tributaries requiring treatment. Participants must be 18 years old or older. Exclusion criteria are current deep vein thrombosis, recurrent varicose veins, arterial disease (ankle brachial pressure index < 0.8), vein diameter <3 mm, unwillingness to participate, inability or unwillingness to complete the questionnaires or attend follow-up appointments, preference for one of the treatment options or currently enrolled in a study of varicose vein treatments.

### Interventions

**Fig. 1:**
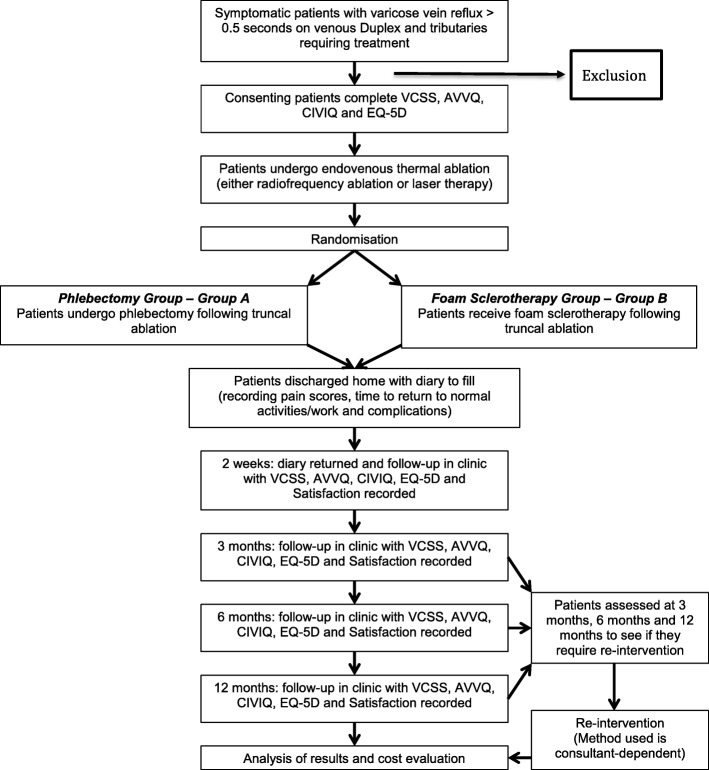
Trial flowchart. AVVQ Aberdeen Varicose Vein Questionnaire, CIVQ-14 Chronic Venous Insufficiency Questionnaire, EQ-5D EuroQol Five-Domain Utility Index, VCSS Venous Clinical Severity Score

All eligible patients will be comprehensively informed about the study and given a written information sheet. If they agree to take part in the study, they will be asked to sign a consent form. Following study enrolment, patients will be randomised to receive either of the two study interventions (AP or FS) to treat their varicose tributaries after having their truncal saphenous veins treated (Fig. 1). At the time of randomisation, each patient will then be allocated a unique study number to maintain confidentiality.

At the baseline, basic demographic data will be collected for each patient. Patients’ contact details will also be collected to enable the scheduling of follow-up appointments. At the pre-intervention evaluation, patients will be asked to complete the following QoL questionnaires: the EuroQol five-domain utility index (EQ-5D), the Aberdeen Varicose Vein Questionnaire (AVVQ) and the Chronic Venous Insufficiency Questionnaire (CIVIQ). Their VCSS will be also assessed.

Following treatment of the truncal saphenous vein, adjuvant AP or FS will be carried out. AP is performed under either a local or a general anaesthetic, usually with a vein hook to enable venous extraction through micro-incisions [12, 13]. FS is guided by an ultrasound scan according to Tessari’s method to create a stable and compact sclerosant foam [14, 15]. Sclerosants used include sodium tetradecyl sulphate and polidocanol. This study will utilise sodium tetradecyl sulphate.

Post-intervention, all patients will be given a diary in which they will be asked to record their post-procedural pain every day for 10 days using a validated 100-mm visual analogue scale and asked to record when they are able to return to their normal daily activities and to work. Verbal and written instruction will be given on how the diary should be completed. In both groups, patients will be asked to wear compression stockings (class II 18–24 mmHg) for 1 week post-treatment and they will be advised to be active as much as possible. Each patient’s general practitioner will be sent a letter informing them of their patient’s participation in the study.

### Primary outcome

The primary outcome of this study is the re-intervention rate at 12 months.

### Secondary outcomes

The secondary outcomes include:

- QoL scores at baseline, 2 weeks, 3 months, 6 months and 12 months using the EQ-5D, AVVQ and CIVIQ-14
- The clinical change in the VCSS from the baseline at 2 weeks, 3 months, 6 months and 12 months
- The degree of bruising and phlebitis at 2 weeks (based on a clinical examination)
- The pain score over the first 2 weeks
- Visual (cosmetic) appearance at baseline, 2 weeks, 3 months, 6 months and 12 months
- The time taken to return to work and normal activities
- Patient satisfaction at 2 weeks, 3 months, 6 months and 12 months
- Cost-effectiveness

### Sample size and study duration

From the published literature, we anticipate a re-intervention rate of 5% in the AP group and 25% in the FS group. Utilising Pearson’s chi-squared test, with a power of 80% at the 5% significance level, 58 patients per group (for a total of 116) would be required to detect an absolute difference of at least 20% in the re-intervention rates. ﻿Assuming﻿ a drop-out rate from follow-up of about 30%, we estimate that 160 patients would be required in total. It is assumed that at least three eligible patients will be enrolled per week, giving a total of 156 patients who could potentially be randomised over the year. With the 12 months of follow-up, this study will run for 24 months.

### Recruitment

All patients referred for treatment of symptomatic varicose veins will be included if they also have primary truncal vein incompetence and visible symptomatic varicosities needing treatment. Potential patients will be identified at outpatient clinic appointments and given a patient information sheet.

### Follow-up treatment periods

**Fig. 2:**
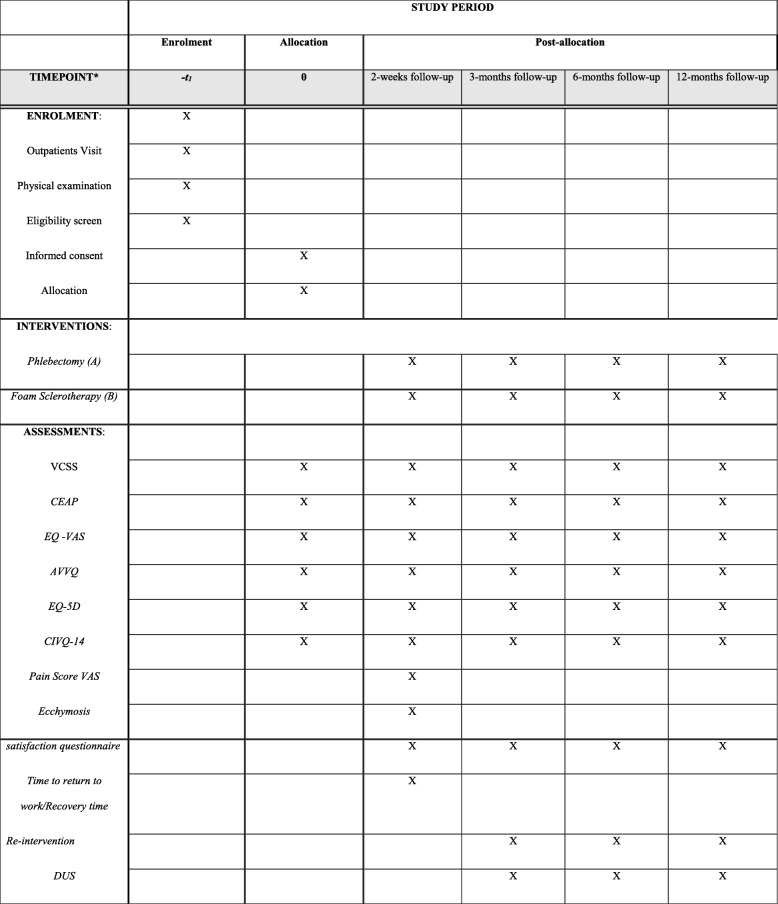
Schedule of enrolment, interventions and assessments

For this research, patients will be seen in the research clinic for follow-ups at 2 weeks, 3 months, 6 months and 12 months (Fig. 2). At the 2-week follow-up, the patient’s diary will be collected, but no decision regarding re-treatment will be taken. At all follow-ups, they will undergo a clinical assessment and their VCSS will be recorded. Additionally, patients will be asked to fill out the EQ-5D, AVVQ and CIVIQ-14 questionnaires. All patients will be asked how satisfied they were with their treatment using a satisfaction questionnaire (ranging from 0 = very bad to 10 = excellent). At the 3-, 6- and 12-month follow-ups, a duplex ultrasonographic scan will be carried out to determine if there is occlusion of the treated vein.

All participants will be sent reminders by post or contacted over the telephone to attend the follow-up visits. If they fail to attend a scheduled visit, the QoL questionnaires will be sent by post to be completed.

### Re-intervention

No decision regarding re-intervention will be made before the 3-month follow-up. From this point, any recurrence of tributaries will be assessed from the patent’s history and a physical examination at the 3-, 6- and 12-month follow-ups. Re-intervention is indicated by the symptomatic recurrence of varicosities in the areas previously treated that are deemed to necessitate treatment by both the clinician and the patient. The method used for re-intervention will be chosen by the clinician who is caring for the patient.

### Randomisation

Signed written consent forms are collected on the day of treatment. Patients will then be randomised into one of the two possible treatment options by equal randomisation using an online computerised web system (SealedEnvelope, London, UK).

### Blinding

After randomisation, at the point of the intervention, neither the patient nor the clinical team will be blinded to the allocation due to the nature of the intervention. However, following treatment, the clinicians conducting the follow-up reviews and the duplex ultrasound scan will be blinded to group allocation.

### Data collection and confidentiality

All patient data will be stored in a password-protected Access database on a password-protected NHS computer at Imperial College London under the guidelines of the Data Protection Act 1998 and the EU General Data Protection Regulation 2016. Patient details will be anonymised as each participant will be allocated a study number. The codes for the allocated study numbers will be kept in the same database. The chief investigator will preserve the confidentiality of participants taking part in the study and is registered under the Data Protection Act.

Patient details, including contact information, will also be recorded on paper forms, such as the diary, questionnaires and clinical scoring sheets. These will be kept in a locked filing cabinet in a locked office for vascular research within the Vascular Surgery Section on Floor 4 North at Charing Cross Hospital (university office) for 10 years in accordance with Imperial College London’s policy. Each patient’s contact details will be discarded once they have been advised of the findings of the study (within approximately 6–12 months following completion of the study) Additional file 1.

### Publication of data

The findings from this study will be presented locally within the hospital, published in a peer-reviewed journal and presented at national and international conferences.

### Statistical analysis

Data collected from each patient will be entered into and analysed using the statistical software package SPSS version 24 (IBM, Armonk, New York, USA), STATA 15SE (STATACorp, College Station, Texas, USA) and Wizard Pro v1.9.29 (Evan Miller, Chicago, USA).

All analyses will be carried out on an intention-to-treat basis. To determine the normality of the data, both a visual test and a Shapiro–Wilk test will be used. If the data are normally distributed, the mean and standard deviation will be reported, otherwise the median and interquartile range. Categorial variables will be presented as frequencies and percentages. If the data are normally distributed, *t*-tests will be used or the Mann–Whitney *U* test if not. Assessments of the changes in scores from the baseline to each follow-up visit will be investigated using a repeated measures ANOVA. The primary outcome measure is a comparison of the re-intervention rates between groups at 12 months. This will be assessed with a Mann–Whitney *U* test.

The data will be assessed for missing values. The complete data set will be analysed using the listwise deletion methodology. The multiple imputation methodology will be used for missing data points.

Freedom from re-intervention curves will be analysed utilising survival analysis and Kaplan–Meier estimation methods with re-intervention classified as the failure event. Missing values for re-intervention due to loss to follow-up will be censored in the survival analysis.

### Cost-effectiveness analysis

We will record equipment costs, the time for personnel to perform the interventions and the cost of the operating theatre. Time to return to work and the QoL gain following the procedure will be also assessed. The cost-effectiveness analysis will be undertaken with the aid of a specialist health economist.

### Data monitoring, safety and quality control

An adverse event is defined as any undesirable medical experience by a patient, whether or not it is related to the intervention. A severe adverse event (SAE) is a life-threatening experience. All adverse events should be documented and these reporting procedures should be followed. Queries should be directed to the chief investigator in the first instance. The chief investigator should be notified of all SAEs within 24 h. All SAEs should be reported to the research ethics committee if, in the opinion of the chief investigator, the event was related to the intervention. Reports of related and unexpected SAEs should be submitted within 15 days by the chief investigator, using the National Research Ethics Service SAE form for non-investigational medicinal product studies. Local investigators should report any SAEs as required by their local research ethics committee, the sponsor, and the research and development office. Imperial College London has public liability insurance to cover negligent harm and non-negligent harm arising from participation in this study. The study will be monitored and audited according to the policies of the Joint Research Compliance Office of Imperial College London.

## Discussion

Despite AP or FS being used worldwide to address varicose vein tributaries, the relative effectiveness of each intervention is still unknown. We aim to perform a randomised controlled trial comparing AP with FS to investigate the difference in the re-intervention rates for the treatment of varicose vein tributaries.

## Additional file

Additional file 1: Trial Spirit 2013 Checklist. (DOC 125 kb)

## Data Availability

Anonymised study data will be available on request and will be uploaded to a secure research server in Imperial College London. All data analyses and manuscripts produced will be available on request.

## Abbreviations

AP: Ambulatory phlebectomy
AVVQ: Aberdeen Varicose Vein Questionnaire
CIVQ-14: Chronic Venous Insufficiency Questionnaire
EQ-5D: EuroQol Five-Domain Utility Index
FS: Foam sclerotherapy
QoL: Quality of life
SAE: Severe adverse event
VCSS: Venous Clinical Severity Score
